# Fiber Optic Impact Location System Based on a Tracking Tandem Low-Coherence Interferometer

**DOI:** 10.3390/s23020772

**Published:** 2023-01-10

**Authors:** Petr Volkov, Andrey Lukyanov, Alexander Goryunov, Daniil Semikov, Evgeniy Vopilkin, Stanislav Kraev

**Affiliations:** The Institute for Physics of Microstructures RAS, Academicheskaya Str. 7, 603087 Afonino, Nizhny Novgorod Region, Russia

**Keywords:** fiber optic sensor, Fabry–Pérot interferometer, acoustic emission

## Abstract

This study proposes a method for detecting small-length fluctuations for fiber-optic sensors (FOS). The method is based on a tracking tandem low-coherence interferometer and enables the ability to compensate for temperature and deformation drifts in FOS. As a result, the constant high sensitivity of FOS over a wide frequency range is guaranteed. Sensitivity to the level of 2 nm in the frequency range of 200 kHz has been demonstrated. The operation of the circuit is demonstrated on the example of the 2D location of acoustic signals using a correlation algorithm for signal processing, known as the time reversal method. It is shown that this system enables us to determine the place of the impact on the sample under the test with an accuracy of about 2 cm using a single sensor.

## 1. Introduction

Acoustic-emission (AE) techniques have been utilized in different applications for structural health monitoring [1]. One of the important tasks is the detection of the shock impacts on a controlled object. Additionally, it is often necessary to define the coordinates of the impact. The most popular way of defining the impact location is through triangulation, based on the measurement of the difference in signals’ arrival times for the sensors located at the edges of a controlled object [2,3,4]. The main problem of this method is that it is necessary to place sensors on all sides of the controlled object. At the same time, the accuracy near the perimeter of the controlled zone is low. Due to the rather high speed of a sound and small times of signal delays for the objects of the actual sizes, this method requires signals with sharp fronts, otherwise its accuracy strongly falls. There is another method for impact location, the so-called “time reverse” method [5,6]. However, this method demands high stability, linearity, and a wide frequency range of the sensors, therefore it was realized with piezoelectric sensors but there have been problems with FOS realization. In this work, we propose the high-stability fiber-optic sensing system for 2D location using the principles of a time reverse method.

Currently there are many variants of FOS for various physical quantities. The most popular are the optical Fibre Bragg Gratings (FBG) and Fabry–Pérot interferometers. The main advantage of FBG is quite simple manufacturing and spectral encoding of the measured parameter [7,8]. However, direct spectral reading is slow and does not allow the use of FBG for acoustic signal detections. There are various methods for reading high frequency oscillations using FBG [9,10], but they have problems with sensitivity and linearity of response. In addition, FBG has a high sensitivity to temperature and deformation [11]. This greatly complicates the use of high-frequency demodulation methods, since all these methods are based on work on the slope of the spectral response of the FBG, but deformation and temperature lead to a big shift of the entire spectral curve of the FBG. So, for acoustic detection, the Fabry–Pérot sensors are usually used [12,13,14,15]. Generally Fabry–Pérot FOS found many applications for detecting various physical quantities, such as temperature, pressure, etc. [16,17,18,19]. The working principle of such sensors is based on the change of their optical length at external influence, for example in temperature sensors, the temperature dependence of the index of refraction [20] and in pressure and vibration sensors, the membrane shift [21,22]. This change is detected further by means of the optical system of registration. There are various schemes of registration for Fabry–Pérot sensors. One of the popular methods is the use of a broadband source and a spectrometer in which a change of the sensor length leads to the shift of the maxima and minima in the spectrum of the reflected light [15,23]. A drawback of such a scheme is a rather low rate of the spectrometer: 1–10 kHz. It enables the use of this variant for slow-changing processes, such as temperature and pressure, but is not suitable for the detection of fast processes, such as, for example, acoustic waves. Instead of a broadband source and a spectrometer, it is possible to use the monochromatic laser with wavelength scanning [24]. In terms of signals processing, this scheme is completely the equivalent to the scheme with a spectrometer, but allows us to make a system more compact and inexpensive. However, in terms of measurement rate, it is, practically, about the same.

Another popular method is laser interferometry, where a coherent monochromatic source and a single photodetector are used. Sensor length variations lead to varying the phase shift between the waves reflected from front and back mirrors. Thus, the intensity of the reflected light varies. Despite the simplicity of this scheme, its main shortcoming is the sensor optical length drift due to temperature change or mechanical deformation. As a result, at the registration of small fluctuations, the sensitivity of the system will change in a random way that will make measurements impossible.

There are various ways of working point drift compensations. One of the most known is the method of homodyne demodulation [25,26,27,28], allowing the complete exclusion of the working point drift. However, in this case, the sensitive element should be placed directly inside the interferometer that strongly complicates the multiplexing and monitoring of remote objects. Another technique for resonator length control is so-called tandem low-coherent interferometry (TLCI) [19,22,29]. This method has high precision and stability of measurements, but has rather low speed. In this work, the original method based on the tracking low-coherent interferometer with a high sensitivity and measurement rate is provided.

## 2. Methods

**Figure 1:**
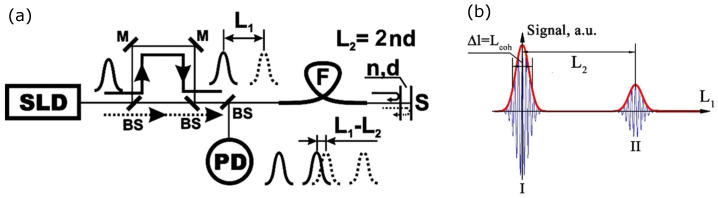
Tandem low-coherence interferometry: (**a**) TLCI scheme. (**b**) Form of a signal in the TLCI system: SLD—superluminescent diode, BS—beamsplitter, M—mirror, F—optical fiber, S—sensor, PD—photodetector; n,d—the refraction index and length of sensor, $L_{1,2}$—optical delays in the reference interferometer and sensor, respectively.

The proposed system is based on the low-coherence interferometry, as shown in Figure 1a.

**Figure 2:**
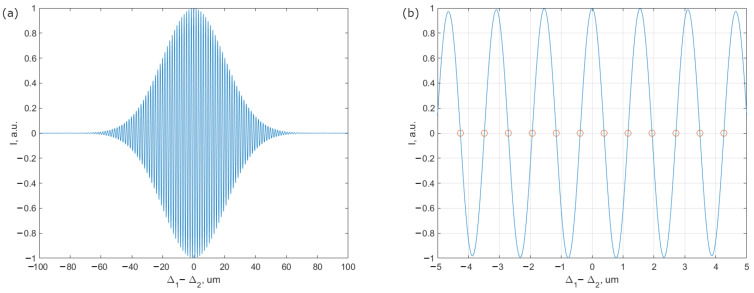
Low coherence signal: (**a**) full signal near $|\Delta_{1}-\Delta_{2}|\approx L_{coh}$. (**b**) Expanded signal with marked points of maximum sensitivity (red circles).

The intensity at the output of the scheme can be described as:

(1) $$I\left(\Delta_{1},\Delta_{2}\right)=\frac{1}{4}(1+\gamma \left(\Delta_{1}\right)+\gamma \left(\Delta_{2}\right)+\frac{1}{2}\gamma \left(\Delta_{1}+\Delta_{2}\right)+\frac{1}{2}\gamma \left(\Delta_{1}-\Delta_{2}\right),$$

where $\Delta_{1,2}$ is the optical path differences in the reference and sensor interferometers and γ(Δ) is the light source autocorrelation function. The form of the signal is shown in Figure 1b. If the power spectral density of the light source has the Gaussian form:

(2) $$G\left(\nu \right)=\frac{1}{\sqrt{\pi {\left(\Delta \nu \right)}^{2}}}\exp\frac{{\left(\nu -\nu_{0}\right)}^{2}}{{\left(\Delta \nu \right)}^{2}},$$

which is quite well performed for the superluminescent diodes, the autocorrelation function will have the form:

(3) $$\gamma \left(\Delta \right)=\exp\left(-\frac{\Delta^{2}}{{L_{coh}}^{2}}\right)\cos k\Delta ,$$

where $L_{coh}=\frac{c}{\pi \Delta \nu }$ is the light source coherence length. It can be seen from (1) that when $|\Delta_{1}-\Delta_{2}|<L_{coh}$ the interference signal will have the form as shown in Figure 2.

Let us call the interferometer—I, the reference interferometer—RI, the interferometer—II, and the sensor interferometer—SI. Thus, we can divide the SI length change into two parts: the slow one which resulted from the temperature and deformation drifts and the fast changes which resulted from the acoustic waves. Usually, the temperature and deformation drifts are in the range of 0–100 Hz and the acoustic frequencies caused by the impact are higher than 1 kHz.

Thus, the optical length of the SI can be described as:

(4) $$\Delta_{2}=\Delta_{2slow}+\Delta_{2fast},$$

where $\Delta_{2slow}$ is the slow drift of the SI and $\Delta_{2fast}$ is the high frequency acoustic waves. We would like to note that this division for fast and slow processes can be varied due to concrete conditions. The main proposed idea is to compensate for the slow drift ($\Delta_{2slow}$) of the SI with the continuous adjustment of the RI ($\Delta_{1}$). If this adjustment will provide at any time the condition

(5) $$k(\Delta_{1}-\Delta_{2slow})=\pi /2\pm \pi n,$$

where *n* ∈ N, then the intensity modulation at the output of the scheme will be determined only by the high-frequency component of the SI modulation ($\Delta_{2fast}$). Graphically, the points where this condition is realised are marked by the red circles in Figure 2b.

Let us add the additional sinusoidal modulation in RI:

(6) $$\Delta_{1}=\Delta_{1slow}+\Delta_{m}\cos\omega_{0}t,$$

where $\Delta_{1slow}$ is the slow adjustment of the RI, $\Delta_{m}$ is the amplitude of the modulation of the RI, and $\omega_{0}$ is the frequency of the modulation of the RI. The modulation frequency should be large in comparison with drift frequencies. Then, the condition (5) will take the form

(7) $$k(\Delta_{1slow}-\Delta_{2slow})=\pi /2\pm \pi n.$$

Let us sign, in more detail, the interference signal in the points of maximum sensitivity (red circles in Figure 2b. For small acoustic signals, when $|\Delta_{2fast}|,\Delta_{m}<<\lambda ,$ the contrast of the interference signal can be taken as a constant. Then, the interference term of the TLCI (term 3 in (1)) will be

(8) $$I_{∼}=A\cos k(\Delta_{1slow}-\Delta_{2slow}+\Delta_{m}\cos\omega_{0}t-\Delta_{2fast}),$$

where *A* is some coefficient determined by reflections in RI and SI. Using standard trigonometric formulas and the well-known Jacobi–Anger expansion [30], Equation (8) can be transformed to:

(9) $$\begin{array}{c} I_{∼}=A\cos\left(k(\Delta_{1slow}-\Delta_{2slow}-\Delta_{2fast})\right)\left[J_{0}\left(k\Delta_{m}\right)+2\sum_{n=1}^{\infty }{\left(-1\right)}^{n}J_{2n}\left(k\Delta_{m}\right)\cos\left(2n\omega_{0}t\right)\right]+\\ +A\sin\left(k(\Delta_{1slow}-\Delta_{2slow}-\Delta_{2fast})\right)\left[2\sum_{n=1}^{\infty }{\left(-1\right)}^{n}J_{2n-1}\left(k\Delta_{m}\right)\cos\left(\left(2n-1\right)\omega_{0}t\right)\right]. \end{array}$$

In cases when the acoustic signal is absent ($\Delta_{2fast}=0$) and the condition (7) is met, it can be seen that the amplitude of the even harmonics of the modulation frequency $\omega_{0}$ turns to zero while the amplitudes of the odd harmonics reach the maximum. Thus, the amplitude of the second harmonic of the $\omega_{0}$ can be used as a feedback signal to track the drift of the SI by tuning RI. In this case, if $\Delta_{m}\ll lambda$ the output intensity modulation at the frequency $\omega_{0}$ will be

(10) $$I_{\omega_{0}}=Ak\sin\omega_{0}t.$$

Since the amplitude of the reference modulation is known, we obtain a calibration signal that allows us to calculate a coefficient for converting the signal modulation from the output of the photodetector into length modulation of the SI.

Let an acoustic signal now be present, i.e., $\Delta_{2fast}\neq 0$. If the amplitude of the spectral component in the acoustic signal at the frequency of $2\omega_{0}$ is small compared to $\Delta_{m}$, then the adjustment of the RI to maintain the condition (7) will also be possible. In this case, the output intensity will take the form:

(11) $$\begin{array}{c} I_{∼}=A\cos\left(k\Delta_{2fast}\right)\left[2\sum_{n=1}^{\infty }{\left(-1\right)}^{n}J_{2n-1}\left(k\Delta_{m}\right)\cos\left(\left(2n-1\right)\omega_{0}t\right)\right]+\\ \;+A\sin\left(k\Delta_{2fast}\right)\left[J_{0}\left(k\Delta_{m}\right)+2\sum_{n=1}^{\infty }{\left(-1\right)}^{n}J_{2n}\left(k\Delta_{m}\right)\cos\left(2n\omega_{0}t\right)\right]. \end{array}$$

In (11), the amplitudes of even harmonics are zero due to the feedback system that provides the condition (7). Thus, if $|\Delta_{2fast}|,\Delta_{m}\ll \lambda$, the (11) can be simplified to

(12) $$I_{\omega_{0}}=Ak\Delta_{m}\sin\omega_{0}t+Ak\Delta_{2fast}+A\left[2\sum_{n=2}^{\infty }{\left(-1\right)}^{n}J_{2n-1}\left(k\Delta_{m}\right)\cos\left(\left(2n-1\right)\omega_{0}t\right)\right].$$

In (12), the amplitude of the first harmonic can be filtered by a narrow band filter and afterwards can be used to calculate the calibration coefficient from the Volts at the photoreceiver output to the nm of the SI variation. The upper odd harmonics can also be filtered by the narrow-band filters but, at the same time, their amplitudes quickly drop with an increase in the harmonic number. Taking into account the small amplitude of modulation ($\Delta_{m}\ll \lambda$) in reality, harmonics with a number greater than 3 are below the noise level.

As a result the output signal can be taken as

(13) $$S_{detected}=\frac{I_{without\;\omega_{0}}}{AI_{\omega }}=\frac{Ak\Delta_{2fast}}{Ak\Delta_{m}}=\frac{\Delta_{2fast}}{\Delta_{m}}.$$

The final signal in (13) does not depend on either the intensity of the light, including on losses in the optical fiber, nor on the interference contrast, nor on the characteristics of the amplifier of the photodetector. At the same time, due to the presence of a calibration modulation signal at the first harmonic, it allows us to obtain absolute values of the sensor length changes. As a result, the proposed approach allows us to combine all the advantages of a tandem low-coherence circuit and the high-coherence schemes: the ability to remove the sensor element from the reference interferometer by an almost arbitrary distance, the insensitivity of the circuit to disturbances along the fiber path, and the high sensitivity.

## 3. Experiment

**Figure 3:**
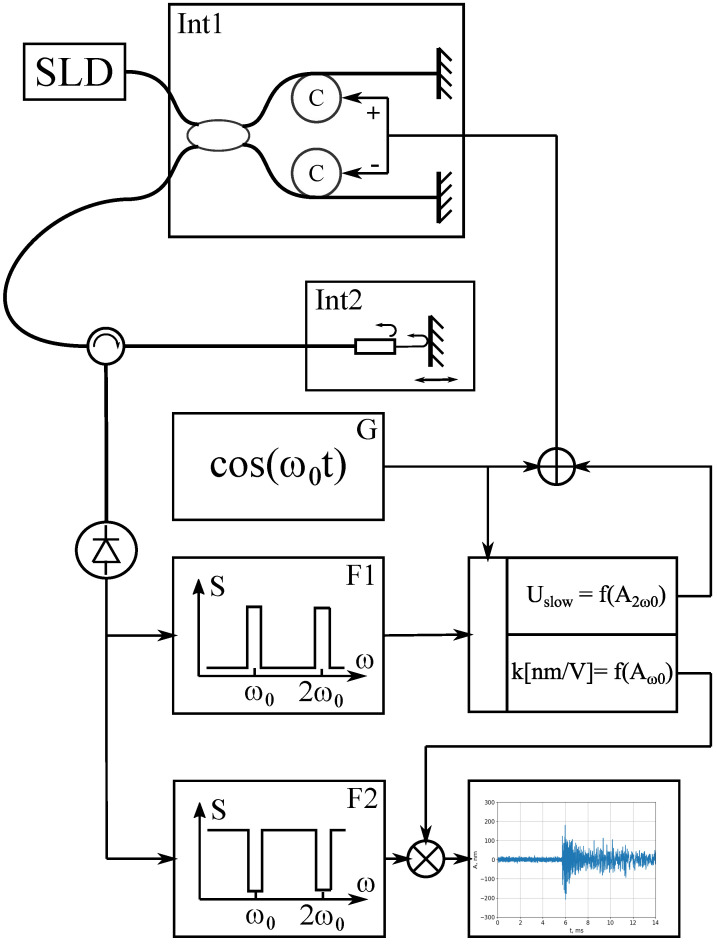
Detection circuit. SLD—superluminescent diode, Int1—reference interferometer, Int2—sensor interferometer, G—generator, F1—bandpass digital filter, F2—bandstop digital filter.

The scheme of the registration is shown in Figure 3:

Light from the superluminescent diode (wavelength 1310 nm, spectral width 40 nm, optical power 1 mW) emits into the fiber-optic Michelson interferometer (Figure 3). There are two piezoceramic coils in the interferometer with optical fiber wound around them. Thus, the optical path difference can be changed by applying voltage to the coils. Then, through the circulator, light enters the sensor and after reflection from the sensors goes to the photodetector. Then, the signal from the photodetector is processed by a personal computer. The digitized signal is passed into two bandpass filters. Signals of the first and second harmonics are extracted in filter F1. The calibration coefficient is calculated by the signal on the first harmonic (refer to (10)). The feedback signal is generated using the second harmonic amplitude. It shifts the RI to the point of maximum sensitivity, in which the condition (7) is fulfilled.

**Figure 4:**
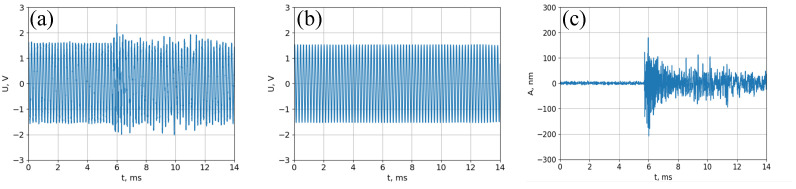
Signal processing. (**a**) Full signal at the output of the photodetector. (**b**) Filtered first harmonic of modulation frequency. (**c**) The detected acoustic signal.

In filter F2, the first three harmonics of the modulation frequency are cut out from the signal by narrow-band filtration, thereby avoiding distortion of the detected signal. The signal processing stages can be seen in Figure 4.

It can be seen from Figure 4 that the sensitivity of the scheme is about 1 nm at the frequency bandgap of about 200 kHz (defined by the photodetector).

**Figure 5:**
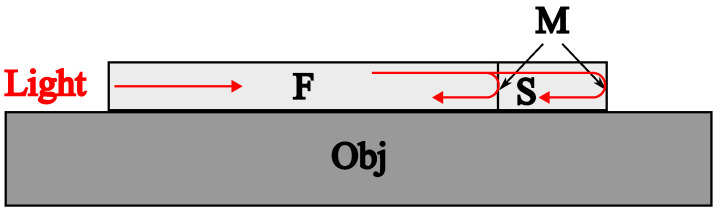
Fiber-optic sensor. F—optical fiber, Obj—object under the test, S—sensor, M—mirrors.

Thanks to the features described above, the proposed scheme made it possible to realize an impact location system (2D) with a single sensor. The in-fiber Fabry–Pérot sensor was used. The scheme of the sensor and its application to a sample under control is shown in Figure 5.

The sensor manufacturing was performed as follows. A layer of the metal (Ni, 40 nm) was deposited onto the fiber tip. After splicing with another fiber, the transparent mirror (reflectivity of about 10%) at the place of the splicing was formed. Afterwards, the fiber was cleaved at the desired length (7 mm in our case). At the final step, a 60 nm layer of the Ni was deposited onto the fiber tip. The resulting resonator was attached to the surface of the tested sample with glue.

**Figure 6:**
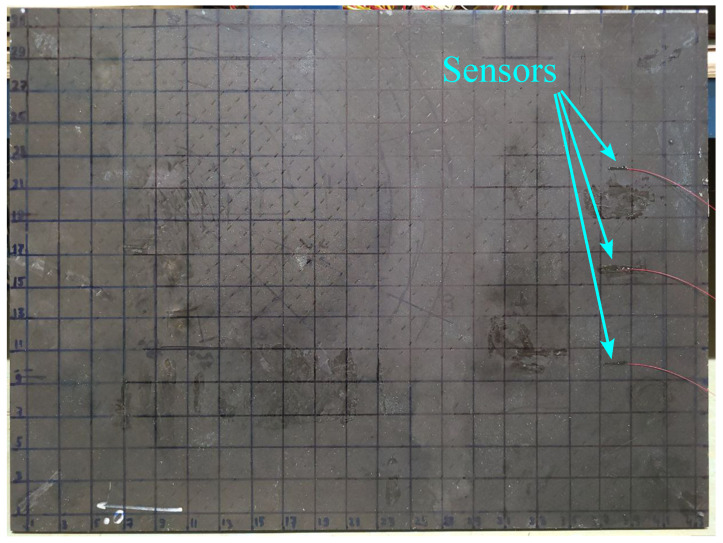
Tested sample.

The tested sample was a 43.5 cm × 32 cm × 5 mm plate made of composite material (Figure 6).

The impact was localized using the so-called “time reverse” algorithm [4,5]. This approach enables us to localize the impact with a single sensor, which is possible due to two reasons: material inhomogeneities and/or re-reflections within the sample, which create a unique configuration of imaginary sources.

**Figure 7:**
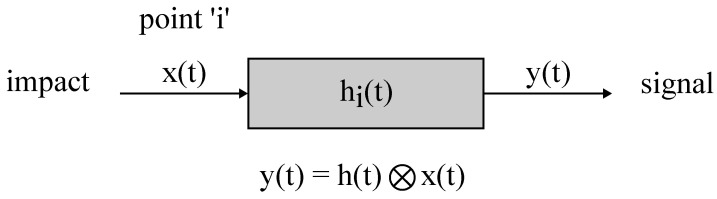
Signal propagation from the point i.

The time reversal method refers to correlation methods. The general idea is as follows. Wave track from the impact point to the sensor can be considered as a linear system with some impulse response $h_{i}\left(t\right)$, where i is the number of the point on the object surface. Thus, in the case of some impact, a signal on the sensor will be defined as a convolution of the impact function with the impulse response (Figure 7).

**Figure 8:**
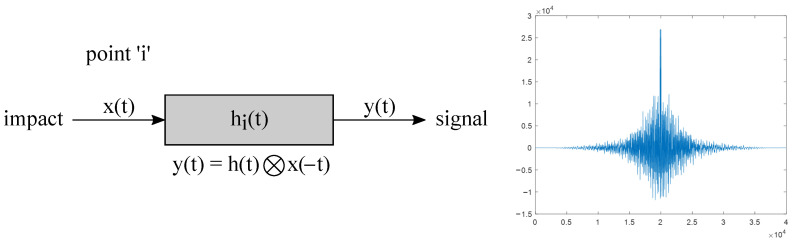
Virtual time reverse experiment.

If we reverse the recorded signal in time and simultaneously reverse the source and receiver places, then a signal with a characteristic maximum will be recorded (Figure 8).

**Figure 9:**
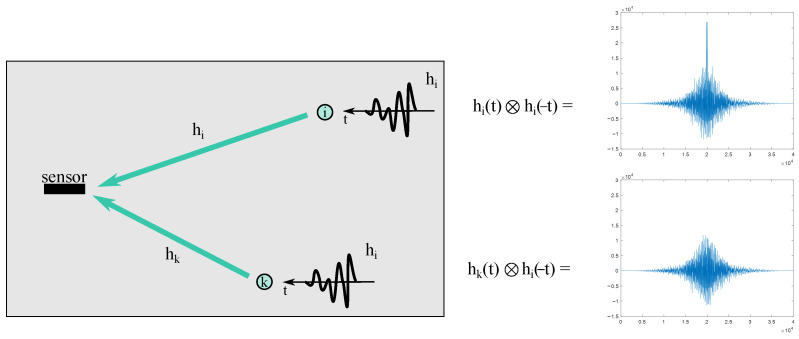
Time reverse selectivity.

This maximum will not be observed if another point-to-sensor path is used (Figure 9). Therefore, if the impulse responses of all calibration points on the plate are known then the position of the unknown impact can be determined by this convolution operation.

## 4. Results

**Figure 10:**
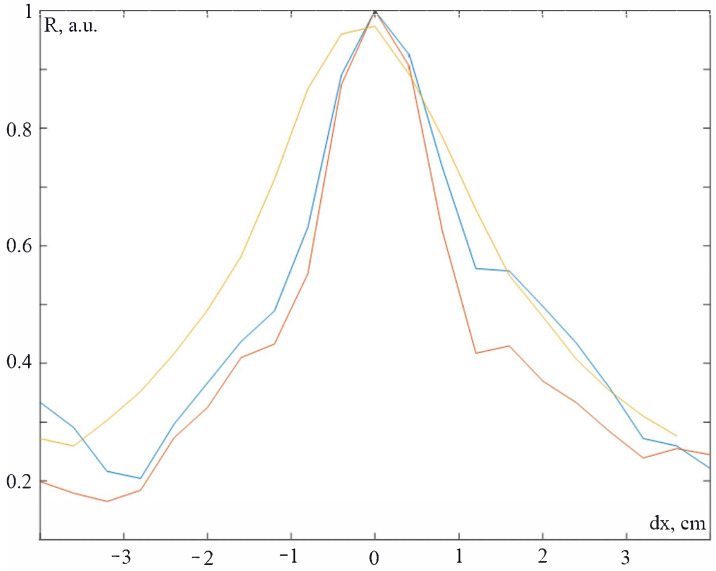
Dependence of the correlation maximum for 3 different points (different colors refer to different points) vs shift of the impact from the calibration point.

As the first step, we have to record the impulse responses for points with some steps. It is obvious that the number of points will be $N=N_{x}N_{y}=\frac{L_{x}}{dl}\frac{L_{y}}{dl}$, where $L_{x,y}$ is the lengths of the object and dl is the step of the grid. Thus, dl should be taken as much as possible to reduce the number of calibration grid points. In Figure 10, the dependence of the correlation maximum for three different points vs the shift of the impact from the calibration point is presented.

It can be seen from Figure 10 that the FWHM is about 2 cm. So 2 cm was taken as a grid step and the dataset of the impulse responses $h_{i,j}\left(t\right)$, where i,j indices were along x and y axes, was written. Afterwards, the impact at the arbitrary point was made. The acoustic signal of the impact S(t) was recorded by the system. The correlation coefficients for all points was scored in accordance with (14):

(14) $$R_{i,j}=h_{i,j}⊗S\left(-t\right)=\int_{0}^{t}h_{i,j}\left(\tau \right)S\left(t+\tau \right)\;d\tau .$$

For a correct comparison of $R_{i,j}$ to each other, they were normalized as:

(15) $$R_{i,j}^{norm}=\frac{R_{i,j}}{\int_{0}^{t}h_{i,j}^{2}\left(t\right)\;dt\int_{0}^{t}S^{2}\left(t\right)\;dt}.$$

**Figure 11:**
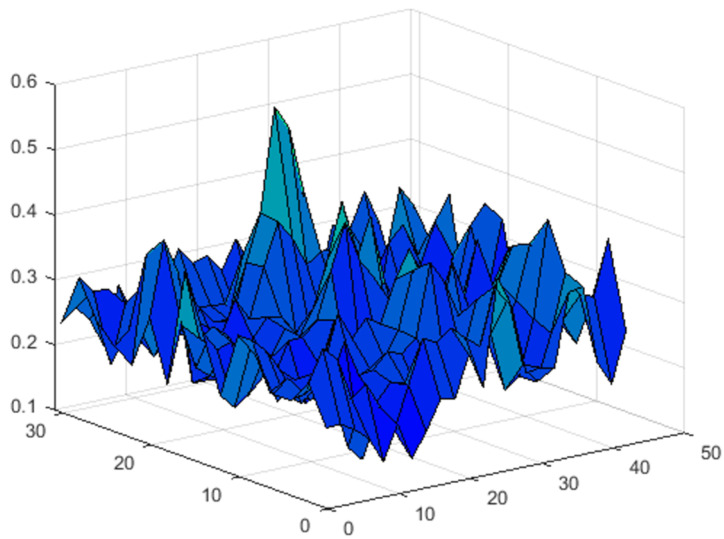
The 2D map of the $R_{i,j}^{max}$ for the point impact for one sensor variant.

Figure 11 shows the surface $R_{i,j}^{max}$, where $R_{i,j}^{max}=max\left(R_{i,j}\right)$ is the maximum of the correlation coefficient for the point i,j.

**Figure 12:**
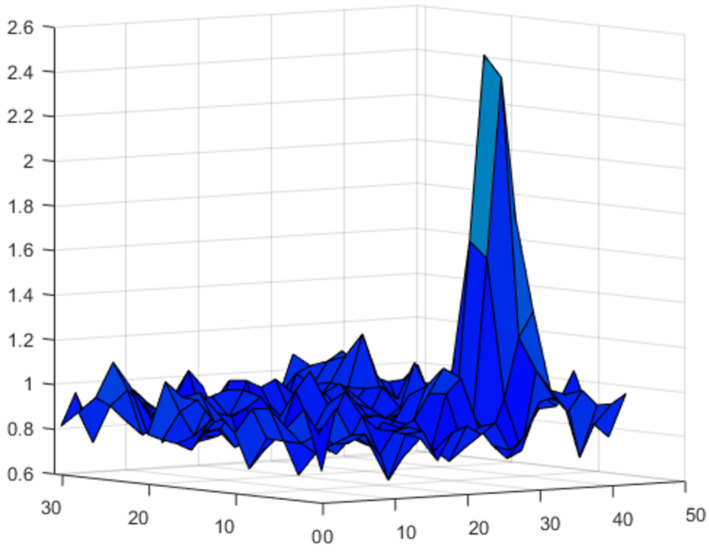
The 2D map of the $R_{i,j}^{max}$ for the point impact for three sensor variant.

Figure 11 clearly shows that the position of the impact can be unambiguously determined by a one-fiber optic sensor. The probability of detection can be increased by increasing the number of sensors. For example, Figure 12 shows the map of the $R_{i,j}^{max}$ for the three sensor system.

Note that, in comparison with the triangulation scheme, here all three sensors are located on one side of the sample at a distance of about 6 cm between them.

**Figure 13:**
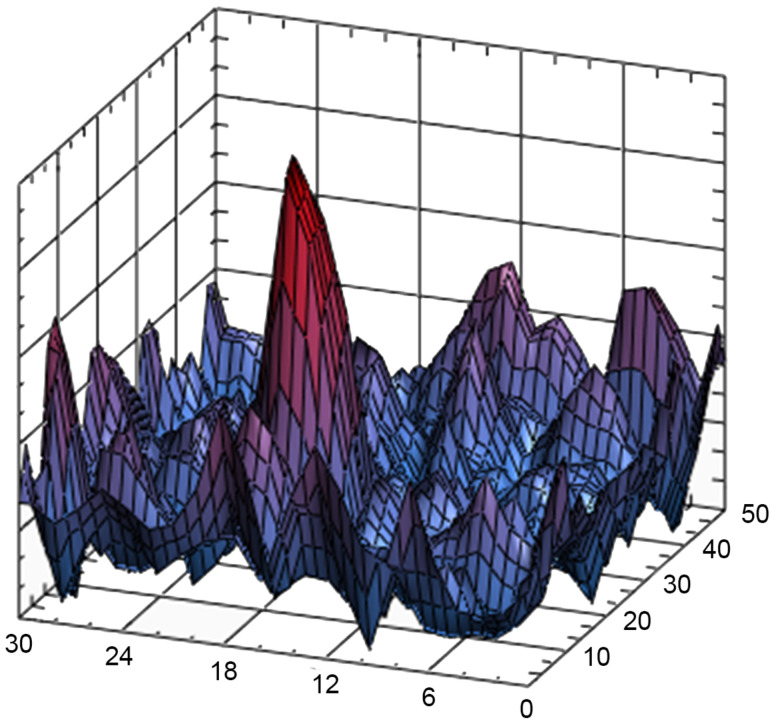
The 2D map of the $R_{i,j}^{max}$ for the finger kick.

In the above experiments, a special striker was used for impact. However, the method and the optical scheme also works with impacts made with different sources. For example, Figure 13 shows the 2D map of the $R_{i,j}^{max}$ for a finger kick (the calibration dataset remain the same in all of the experiments).

Some widening is observed due to the fact that high-frequency components are not excited at the long impact. However, the maximum is still clearly visible.

**Figure 14:**
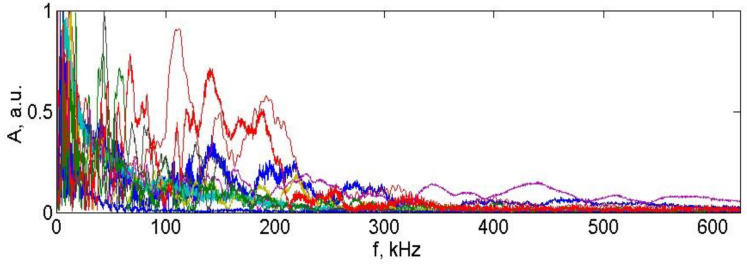
Signals spectrum for 10 different acoustic signals (different colors refer to different signals) recorded by the proposed circuit map of the $R_{i,j}^{max}$ for the finger kick.

The bandwidth of the scheme is determined by two factors: the frequency dependence of the FOS sensitivity and the bandwidth of the photodetector. In our case, the bandwidth of the photodetector was about 200 kHz and was determined by the amplifier circuit. The photodetector bandwidth selection was based on the sampling frequency of the used analog-to-digital converter (ADC), which was 1 MHz, and the characteristics of the signal processing system. At the same time, the frequency range of the FOS sensitivity depends on the ratio of its length and the sound velocity in the material. In our case, in the composite material, the sound velocity was about 1.5 km/s, which corresponds to a wavelength of 15 mm at a frequency of 100 kHz. The length of the sensor was 7 mm in order to have a maximum sensitivity at the frequency of the acoustic signal of about 100 kHz, i.e., in the middle of the frequency range of the photodetector. Obviously, this parameter can be easily changed due to a change in the design of the sensor, for example, by changing its length. Figure 14 illustrates the frequency range of the system by spectra of the 10 different acoustic signals recorded by the proposed circuit.

It can be seen from Figure 14 that the main energy of the signals lies approximately in the 0–200 kHz range.

## 5. Discussion and Conclusions

Currently, fiber-optic sensors are becoming more and more widespread. One of the areas wherein their use looks very promising is the detection of impacts on different objects. At the same time, in such tasks, it is also important to determine the location of the impact. Most often, piezoelectric sensors and triangulation are used for this purpose. Note that the use of fiber-optic systems is difficult in such tasks for two reasons. One of them is the difficulty of detecting frequencies of above 10–20 kHz in standard schemes with a broadband source and spectrometers. This is due to the limited speed of the existing spectrometers. Additionally, coherent detection schemes do not provide the required measurement stability due to sensor slow drifts caused by temperature and slow deformation.

In this work, an original scheme for detecting acoustic waves using fiber-optic Fabry–Pérot sensors was proposed. It is based on the tracking tandem low-coherent interferometer, which allows us to completely eliminate temperature and deformation drifts of the sensor length. The main feature of the proposed scheme is additional modulation outside the sensor, which enables us to control the position of the working point of the entire scheme and, due to the feedback circuit, and maintain it at the point of maximum sensitivity, ensuring constant sensitivity and high linearity of response. Thus, the proposed method combines the advantages of coherent schemes, such as high sensitivity and a wide frequency range, and at the same time completely eliminates the influence of sensor drifts. In most of the well-known methods for sensor drift compensation, the sensing element should be placed inside a measuring interferometer. In the proposed method, the sensing element can be placed at any reasonable distance determined by optical fiber loss from the measuring interferometer. At the same time, the system for sensor drift compensating simultaneously solves the problem of compensating for fluctuations of light intensity caused by the external influence on the optical fiber which connects the sensor and the detection scheme due to automatic built-in calibration.

The sensitivity of the proposed scheme was mainly determined by the excess noise of the used wideband source. In our case, the noise level (3σ) was about 2 nm with a bandwidth of the receiving system (that is, the bandwidth of the photodetector amplifier) of about 200 kHz. Such sensitivity is sufficient to detect most kinds of impacts that can be dangerous to the structure under control. At the same time, the combination of wideband and high sensitivity allows us to save signals with high detail, which increases the stability and the resolution of the correlation methods.

The stability of the proposed system was demonstrated by the implementation of the time reversal algorithm for locating the position of the impact on the plane composite object. The time reverse algorithm is strongly dependent on the linearity of the sensor and the stability of its characteristics. The experiments have shown that a once-taken map of impulse responses allows measurements to be carried out without losing accuracy for various types of impacts over a long time. At the same time, you can determine the impact position over the entire object, even having only one receiving sensor. The lateral resolution was about 2 cm, which is usually enough for the real structures. The very important result—that the map of the impulse responses recorded with a special reference striker—can be used for different types of the impacts.

The proposed results can be important and useful for the structural health monitoring of various objects. Potentially, it allows us to determine not only the fact of the impact and its location, but also its different characteristics, such as time form, energy, and so on. It is also suitable for weak impact, since in the proposed scheme acoustic vibrations are measured in absolute units of displacement with a resolution in the nanometer range.

## Data Availability

All evaluated data are presented in this paper in the graphical form. The raw measured data of this study are available on request from the corresponding author.

## Abbreviations

The following abbreviations are used in this manuscript:

MDPI	Multidisciplinary Digital Publishing Institute
DOAJ	Directory of open access journals
TLA	Three letter acronym
LD	Linear dichroism
TLCI	Tandem low coherence interferometry
RI	Reference interferometer
SI	Sensor interferometer
FBG	Fiber Bragg grating
